# Aspects of Sustainability: Cooperation, Job Satisfaction, and Burnout among Swiss Psychiatrists

**DOI:** 10.3389/fpubh.2015.00025

**Published:** 2015-02-11

**Authors:** Johanna Baumgardt, Jörn Moock, Wulf Rössler, Wolfram Kawohl

**Affiliations:** ^1^Competence Tandem Integrated Care, Leuphana University of Lueneburg, Lueneburg, Germany; ^2^Laboratory of Neuroscience (LIM 27), Institute of Psychiatry, University of São Paulo, São Paulo, Brazil; ^3^University Hospital of Zurich, Zurich, Switzerland; ^4^Department for Psychiatry, Psychotherapy and Psychosomatics, Centre for Social Psychiatry, University Hospital of Psychiatry Zurich, Zurich, Switzerland

**Keywords:** sustainability indicators, cooperation, job satisfaction, burnout, psychiatrists, outpatient mental health care

## Abstract

**Purpose:** Greater sustainability in mental health services is frequently demanded but seldom analyzed. Levels of cooperation, job satisfaction, and burnout are indicators of social sustainability in this field and are of particular importance to medical staff. Because registered psychiatrists play a central role, we assessed the *status quo* and interactions between these three factors among registered psychiatrists in Switzerland.

**Method:** A postal survey with three standardized questionnaires about cooperation, job satisfaction, and burnout was conducted among all registered psychiatrists in the German-speaking part of Switzerland (*n* = 1485). Addresses were provided by the Swiss Medical Association.

**Results:** Response rate was 23.7% (*n* = 352), yielding a largely male sample (62.8%; *n* = 218) aged 55.5 ± 8.7 years old. Quantity (47 ± 56.2 contacts over 3 months) and duration (91.1 ± 101.6 min per week) of cooperation was found to be diverse depending on the stakeholder. Quality of cooperation was greatest in general practitioners (81.5%) while it was worst in community mental health providers (54.9%). Overall job satisfaction was assessed rather high (3.7 ± 0.8), and burnout rates were below crucial values (Emotional Exhaustion, 2.9 ± 0.8; Depersonalization, 1.9 ± 0.5). Both were positively influenced by cooperation. The strongest correlation was found between job satisfaction and burnout, and both had significant inverse relationships in all dimensions.

**Conclusion:** To foster sustainability in outpatient mental health care regarding cooperation, job satisfaction, and burnout, personal aspects such and age or years of registration, organizational aspects, such as networking and practice setting, as wells as supportive aspects such as psychotherapy, and self-help groups, must be considered. Quality of cooperation should be reinforced in particular. Because Integrated and Managed Care models cover several of these factors, the models should be more strongly embedded in health care systems.

## Introduction

Higher sustainability in mental health care is frequently demanded since sustainability is the only concept that takes into account the long-term impact of present actions and analyzes current problems in terms of ecological, economic, as well as social factors with the aim of balancing diverging needs and interests (1). However, at present, there is neither sufficient data on this topic nor a consensus on the definition of a “sustainable health care system” or on what factors characterize such a system (2, 3). Instead, there is a wide range of definitions and approaches, calling for a need to adapt and operationalize appropriate indicators for research in this field (4, 5). In this article, we define – expanded by the ecological dimension – a sustainable health care system as “*a system that is designed to meet the health and health care needs of individuals and the population (*…*); leads to optimal health and health care outcomes; responds and adapts to cultural, social, and economic conditions and demands; and does not compromise the outcomes and ability of future generations to meet their own health care needs*” (p. 8) (6).

With this as a background, current trends in outpatient mental health care show the necessity for research from a sustainable point of view. Thus, the demand for psychiatric services is increasing worldwide because of population growth, greater evidence for the treatability of mental illness, more efficacious medications, and greater social acceptability of mental illness (7, 8). At the same time, there is less money available for, and spent on, the social sector (9) and a global decline in staff. For example, in England, psychiatrists were included on the “national shortage occupation list” (10). Additionally, recruitment is declining (11) as evidenced in the UK where around one in seven posts remained vacant or were filled with locums (12). Furthermore, psychiatrists are aging, as evidenced in Germany, where the proportion of physicians younger than 35 years of age has dropped from 26.6% in 1993 to 18% in 2013 (13). These developments can also be observed in Switzerland (14) where the incidence of psychological disease is between 25 and 30%, about 50% of the residents are affected by psychological problems at least once in their lives (15), and the proportion of medical students specializing in psychiatry fell from 12.4% in 1998 to 4.2% in 2004 (16).

In this study, three indicators of sustainability in health care systems were evaluated. They were cooperation with the various outpatient mental health care providers, an indicator of sustainability on the meso level (17, 18), and job satisfaction and burnout, which are indictors of sustainability on the micro level (19–22). These indicators were chosen for several reasons:

1. To account for the interactions of individual and organizational aspects of health care, we investigated indicators on the micro and meso level.
2. Because people with mental health problems often depend on the full continuum of care, mental health care is mostly characterized by the use of many kinds of medical, nursing, and community mental health services (23, 24). Cooperation, defined as an intentional, temporary, problem-centered, and professionally differentiated form of collaboration with equal rights for all partners who make agreements about particular joint courses of action (25, 26), is thought to be one of the most effective actions for continuity of care as well as high quality care with neither gaps nor excesses in supply (27, 28). Thus, multi-professionalism and cooperation between various services, service providers, and professional groups is not viewed as a choice in modern mental health care, but a basic necessity (29).
3. Job satisfaction is an indicator of sustainability because it belongs to basic human needs and is part of one’s quality of life (19). If poorly developed or absent among mental health care professionals, it can have a substantial impact on quality of care, efficiency of health services, recruitment, and retention of qualified staff (7, 23, 30). Low job satisfaction can lead to a decline in medical graduates (31), increased absenteeism, and high staff turnover (30). To establish and maintain a sustainable outpatient mental health care system, it is therefore important to ensure high job satisfaction among its providers.
4. Burnout, as one form of work-related burden, is an indicator of sustainability that can point to an unhealthy job situation (20). It is related to the number of medical errors (32), patients’ safety (33) and outcomes (34), the type of work environment, and retention of staff (35). Physicians, compared to other professional groups, have a higher prevalence of psychological illness (36), and among them, psychiatrists show higher burnout (37) and the highest suicide rate (38).

Cooperation with other mental health professionals, job satisfaction, and burnout can be regarded as especially relevant for outpatient mental health care providers, e.g., registered psychiatrists. This profession plays a central role in the Swiss outpatient mental health care system because the majority of patients with mental problems seek outpatient therapy (39). Therefore, registered psychiatrists are a focal point for analyzing sustainability. Even though the named indicators cover only specific aspects of the wider issue of sustainability, researching them can help to identify weaknesses in a health care system and provide stakeholders with a good understanding of the factors influencing its sustainability (7, 40). To our knowledge, no studies have been conducted on cooperation, job satisfaction, and burnout among registered psychiatrists in Switzerland jointly from a sustainable point of view. Therefore, we conducted an exploratory survey to determine the *status quo* as well as the influencing factors of relationships between these three indicators of sustainability.

## Materials and Methods

### Recruitment

A postal survey was conducted between July 2012 and February 2013. The sample consisted of 1485 registered psychiatrists in German-speaking Switzerland. Being listed as a registered psychiatrist at the Swiss Medical Association was the inclusion criteria. Because questionnaires were anonymous, follow-up with non-respondents was not possible.

### Questionnaires

Three standardized questionnaires were sent. One questionnaire was used to compare sociodemographic aspects, work characteristics (years as a practicing registered psychiatrist, practice setting, group work status, amount of psychotherapy patients, and amount of chronically ill patients), and duration of cooperation (minutes spent on cooperation per week overall, with medical care, or with community mental health services). It was also used to compare quantity (number of cooperation contacts over 3 months) and quality of cooperation (1, *very good* to 5, *unsatisfying*) with other mental health care providers and organizations. Specifically, these were general practitioners, nursing outreach services, medical specialists (other than psychiatrists), psychotherapists, assisted living institutions, psychiatric or day hospitals, and community mental health service providers (41).To measure job satisfaction, we used the work satisfaction survey in German (42), which contains seventeen items and is divided into six dimensions: Patient Care, Burden, Work-Related Income and Prestige, Personal Rewards, Professional Relations, and Global Item. Answers ranged from 1, *extremely unsatisfied* to 5, *extremely satisfied*. To evaluate burnout, the German version of the Maslach Burnout Inventory for people in Health Services (MBI-D) (43) was used. It consists of 21 items divided into three dimensions: Emotional Exhaustion, Depersonalization, and Personal Accomplishment. Answers ranged from 1, *never* to 6, *very often*. At present, no standardized threshold values for this instrument exist. Values considered as “early detection” ranged from ≥3 to ≥3.5 for the subscales Emotional Exhaustion and Depersonalization; values ≥5 were indicative of burnout that required treatment.

### Statistics

Data analysis using SPSS 21 and descriptive statistics (frequency distributions, mean, SD, and range), interferential statistics (chi-square test, Wilcoxon signed rank test, unpaired *t* test, paired *t* test, analysis of variance, Pearson product moment correlation, and point-biserial correlation) as well as uni- and multivariate linear regression models was carried out. The variables cooperation, job satisfaction, and burnout were the dependent variables; sociodemographic aspects and work characteristics were independent variable. Statistical significance was when *p* values were 5% or less.

## Results

A total of 352 registered psychiatrists replied to the questionnaires, giving a response rate of 23.7%. Five participants working predominantly in a hospital were excluded from further analysis, thereby leaving 347 data sets.

### Sociodemographic and work-related aspects

The majority (62.8%) of participants was male and ranged in age between 51 and 60 years (44.7%). Most of them had been registered between 11 and 20 years (31.2%) and worked in a group practice setting (57.4%; Table 1).

**Table 1:** **Sociodemographic and work-related aspects of respondents**.

Variables	Variable label	Frequencies	%	Mean ± SD	Minimum–maximum
Sex (*n* = 347)	Female	129	37.2	
	Male	218	62.8	
Age (*n* = 347)	≤50	97	28.0	55.5 ± 8.7	36–86
	51–60	155	44.7	
	≥61	95	27.4	
Years as a practicing registered psychiatrist (*n* = 343)	≤10	136	39.7	14.7 ± 9.7	0.3–45
	11–20	107	31.2	
	≥21	100	29.2	
Practice setting/group work status (*n* = 345)	Single practice	147	42.6	
	Group practice with medical practitioners	134	38.8	
	Group practice psychologists	142	41.2	
	Group practice with both	73	21.2	
Amount of psychotherapy patients (*n* = 345)	≤49%	67	19.4	64.3 ± 24.2	2–100
	50–69%	94	27.2	
	70–89%	112	32.5	
	≥90%	72	20.9	
Amount of chronically ill patients (*n* = 343)	≤15%	78	22.7	33.3 ± 21.5	0–100
	16–30%	124	36.2	
	31–45%	48	14.0	
	≥46%	93	27.1	
Referrals to self-help groups^a^ (*n* = 345)	0	103	29.9	
	1–2	164	47.5	
	≥3	78	22.6	
Attendance at regional psychiatric expert meetings^b^ (*n* = 343)	0	56	16.3	
	1–3	160	46.6	
	≥4	127	37.0	

*^a^Over 3 months*.

*^b^Defined in a broad way as all meetings available to registered psychiatrists, e.g., quality circles, meetings of occupational associations, helpers’ conferences, etc*.

### Cooperation

#### Descriptive statistics

Duration, frequency, and quality of cooperation are shown in Table 2. As seen, respondents spent more time on cooperation activities in medical and psychological care (58.1 ± 68.3) than with complementary psychiatric services (35.4 ± 57.9; *p* < 0.001) and showed a wide range of quantity of cooperation (0–590).

**Table 2:** **Descriptive statistics of quality, quantity, and duration of cooperation of respondents**.

	*n*	Mean	SD	Range
**Minutes spent on cooperation per week**
• Overall	337	91.1	101.6	0–700
• With medical and psychological care^a^	336	58.1	68.3	0–600
• With complementary psychiatric services^b^	316	35.4	57.9	0–600
Cooperation contacts over 3 months^c^	339	47.0	56.2	0–590
Quality of cooperation^c^^,^^d^	343	2.1	0.6	

*^a^Hospitals, medical specialists, general practitioners, and psychotherapists*.

*^b^Assisted living institutions, social psychiatric services, counseling centers, and day centers*.

*^c^Mean over all stakeholders questioned: general practitioners, nursing outreach services, medical specialists (other than psychiatrists), psychotherapist, assisted living institutions, psychiatric or day hospitals, and community mental health services*.

*^d^Range: 1 = very good to 5 = unsatisfying*.

**Figure 1:**
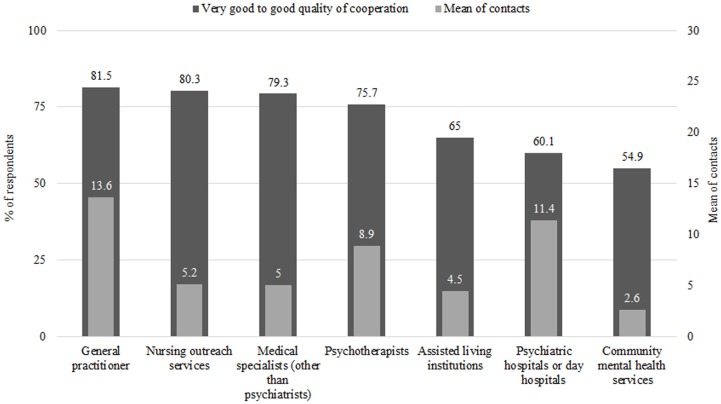
**Comparison of quality and quantity of cooperation**.

An overview of quantity of cooperation compared to the stated quality of cooperation regarding different players of mental health care is given in Figure 1. The highest quality and greatest quantity of cooperation were found in general practitioners; the lowest were found in community mental health providers.

#### Analytic statistics

Significantly more cooperation was found among younger, shorter registered, male psychiatrists with a large number of chronically ill patients and fewer psychotherapy patients (Table 3). More patient referrals and relatives to self-help-groups as well as participation at psychiatric expert meetings correlated positively with greater cooperation.

**Table 3:** **Correlations of sociodemographic and work-related aspects with quantity and duration of cooperation**.

	Amount of contacts over 3 months	Minutes per week		
		Overall	With medical care	With community mental health care
Sex^a^	−0,114*			
Age	−0,201**	−0,209**	−0,175**	−0,147**
Years as a practicing registered psychiatrist	−0,201**	−0,144**	−0,130*	
Amount of psychotherapy patients	−0,293**	−0,193**	−0,113*	−0,201**
Amount of chronically ill patients	0,129*	0,147**		0,207**
Referrals to self-help groups^b^	0,333**	0,195**	0,200**	0,146**
Attendance at psychiatric expert meetings^c^	0,154**			

*Correlation is significant at the 0.05 level (2-tailed).

**Correlation is significant at the 0.01 level (2-tailed).

*^a^0 = male; 1 = female*.

*^b^0 = 0–2 referrals; 1 = ≥3 referrals*.

*^c^0 = 0–3 times participated; 1 = ≥4 times participated*.

### Job satisfaction

#### Descriptive statistics

Results of the Job Satisfaction Survey are shown in Table 4. As seen, the highest scores were found in Patient Care (4.1 ± 0.5) and Personal Rewards (4.1 ± 0.6) while the lowest was in Burden (3.1 ± 0.8).

**Table 4:** **Descriptive statistics of job satisfaction survey**.

	Mean	SD
Patient Care	4.1	0.5
• Relations with patients	4.3	0.6
• Autonomy in treating one’s patients	4.0	0.8
• Autonomy in referring one’s patients to a specialist	4.0	0.9
• Quality of care can be provided	4.0	0.6
Burden	3.1	0.8
• Workload	3.5	1.0
• Time available for family, friends, or leisure	3.4	1.1
• Work-related stress	3.2	0.9
• Administrative burden	2.5	1.0
Income-prestige	3.2	0.9
• Social status and respect	3.4	1.0
• The manner in which one is currently paid	3.1	1.1
• Current income	3.2	1.0
Personal rewards	4.1	0.6
• Intellectual stimulation	4.2	0.8
• Opportunities for continuing medical education	3.9	0.8
• Enjoyment of work	4.2	0.8
Professional relations	3.8	0.8
• Relations with peers	3.7	0.9
• Relations with non-medical staff	3.9	0.9
Global item = satisfaction with current job situation in general	3.7	0.8

*Range: 1 = extremely unsatisfied to 5 = extremely satisfied*.

#### Analytic statistics

Significant correlations were found regarding practice setting, the amount of psychotherapy patients and attendance at psychiatric expert meetings (Table 5). Duration of cooperation correlated negatively with Burden and the Global Item while quantity correlated positively with Professional Relations. Higher quality of cooperation was associated with significantly higher job satisfaction in all subscales but Burden. Multivariate analyses showed no or very small effects in single subscales and are therefore not reported.

**Table 5:** **Correlations of sociodemographic and work related characteristics, cooperation, job satisfaction, and burnout**.

	Job satisfaction^a^						Burnout/MBI-D^b^		
	Patient care	Burden	Income- prestige	Personal rewards	Professional relations	Global item	Emotional exhaustion	Deperso- nalization	Personal accomplishment
**Sociodemographic & work-related characteristics**
Age							−0,123*		
Single practice^c^					−0,143**				
Amount of psychotherapy patients		0,164**					−0,136*	−0,160**	
Referrals to self-help groups^d^		−0,118*	−0,113*						
Attendance at psychiatric expert meetings^e^					0,187**				
**Cooperation**
Minutes per week
• Overall		−0,116*							
• With medical care		−0,186**			0,115*	−0,143**	0,144**	0,107*	
Contacts over 3 months					0,118*				0,132*
Quality^f^	−0,240**		−0,202**	−0,270**	−0,360**	−0,197**			−0,173**
**Burnout/MBI-D**
Emotional exhaustion	−0,211**	−0,634**	−0,363**	−0,361**	−0,116*	−0,474**			
Depersonalization	−0,251**	−0,274**	−0,143**	−0,320**	−0,110*	−0,213**			
Personal accomplishment	0,283**	0,224**	0,142**	0,307**	0,203**	0,297**			

*Correlation is significant at the 0.05 level (2-tailed).

**Correlation is significant at the 0.01 level (2-tailed).

*^a^1 = extremely unsatisfied to 5 = extremely satisfied*.

*^b^1 = never to 6 = very often*.

*^c^0 = no; 1 = yes*.

*^d^0 = 0–2 referrals; 1 = ≥3 referrals*.

*^e^0 = 0–3 times participated; 1 = ≥4 times participated*.

*^f^1 = very good to 5 = unsatisfying*.

### Burnout

#### Descriptive statistics

Results of the MBI-D are shown in Table 6.

**Table 6:** **Descriptive statistics of MBI-D**.

	Mean	SD
Emotional exhaustion	2.9	0.8
• I feel frustrated by my job	2.7	0.9
• I feel burned out from my work	2.6	1.2
• I feel used up at the end of the work day	3.7	1.2
• I feel emotionally drained from my work	3.2	1.2
• Working with people all day is really a strain for me	4.1	1.2
• I feel fatigued when I get up in the morning and have to face another day on the job	2.6	1.1
• Working with people directly puts too much stress on me	2.2	0.9
• I feel like I’m at the end of my rope	1.8	1.0
• I feel I’m working too hard in my job	3.1	1.3
Depersonalization	1.9	0.5
• I worry that this job is harden me emotionally	2.3	1.1
• I don’t really care what happens to some patients	1.6	0.7
• I’ve become more callous toward people since I took this job	1.8	0.9
• I feel uncomfortable about the way I have treated some patients	2.3	0.8
• I feel I treat some patients as if they were impersonal objects	1.3	0.6
Personal Accomplishment	5.0	0.5
• I feel I’m positively influencing other people’s lives through my work	5.0	0.7
• I feel very energetic	4.6	0.8
• I can easily create a relaxed atmosphere with my patients	5.2	0.6
• I can easily understand how my patients feel about things	5.2	0.7
• I have accomplished many worthwhile things in this job	5.0	0.8
• I deal very effectively with the problems of my clients	5.0	0.6
• I feel exhilarated after working closely with my patients	4.8	0.8

*Range: 1 = never to 6 = very often*.

#### Analytic statistics

Greater age (−0.123*), more psychotherapy patients (−0.136*; −0.160**) and higher quantity of cooperation (0.132*) were associated with lower burnout risk, while duration of cooperation (0.144**; 0.107*) was associated with higher burnout risk (Table 5). Higher quality of cooperation was associated with higher rates in Personal Accomplishment. The strongest and most significant interactions were observed between job satisfaction and burnout. Here, higher job satisfaction was associated with lower burnout risk on all subscales. Multivariate analyses showed no or only very small effects in single subscales and are therefore not reported.

## Discussion

This data shows a great range of quantity, duration, and quality of cooperation and those aspects depend on the particular stakeholder that psychiatrists cooperate with. Job satisfaction was rated rather high while burnout risk was low. Both indicators were positively influenced by the quality of cooperation. The strongest and most significant correlations were found between job satisfaction and burnout, which were found to have significant inverse relationships in all dimensions.

Sociodemographic findings of this study are consistent with official statistics, which also indicate a male majority among physicians in outpatient health care and a similar mean age (44). About 57.4% of the respondents were found to be working in a shared practice setting, supporting previous findings that psychiatrists prefer to work in shared venues rather than individually (23). The age range of this study’s participants is noteworthy because it confirms data from Canada (23), but differs from data from Germany (45) and the UK (34), which show that psychiatrists work fewer years. Thus, the early retirement seen in other countries (12, 46) does not seem to be a problem in Switzerland.

Regarding cooperation, our data confirms a German study which also found that a majority of registered psychiatrists refer their patients to self-help groups and frequently attend psychiatric expert meetings. In addition, a great range of quantity and duration of cooperation was found regarding the individual stakeholders. Quality of cooperation was rated mostly positive, especially in the medical professions (45). Nevertheless, in both studies, a great part of the respondents rated their quality of cooperation as sufficient or worse. Differences were seen in the duration of cooperation, where German psychiatrists spent about half an hour more per week compared to their Swiss colleagues. Our study expanded on the German study; we found lower quantities of cooperation among female, older, and longer-registered psychiatrists with a large amount of psychotherapy patients who less-frequently referred patients to self-help groups and rarely attended expert meetings.

The high levels of job satisfaction among psychiatrists confirm data from other German-speaking areas (11, 33, 46, 47), but differ regarding studies from other highly developed countries that state moderate or low rates (40, 48, 49). Finding the greatest satisfaction with personal rewards and the lowest satisfaction with burden was also shown in other studies (42). So far, research shows diverse findings concerning all the factors associated with practitioners’ levels of career satisfaction, except income (49). Factors range from concrete aspects such as stress (48, 50), professional rewards (51), workload, quality of services and facilities available to patients, psycho-social competencies, and organization of work (23) to more general ones such as a good balance between professional and personal issues (7, 47). Data confirm higher satisfaction among psychiatrists who worked in a shared practice setting (23) and who stated they had high quality of cooperation (52). Additionally, it reinforces that psychological treatment for patients and being involved in regular professional exchanges may foster job satisfaction. Diverse findings concerning quantity and duration of cooperation point out that intense cooperation can have positive as well as negative effects.

The low burnout rates found in this study confirm the results of Amstutz et al. (53) and may indicate that Swiss psychiatrists work well within the current mental health care system. Low and medium burnout rates among psychiatrists were also found in Austria (47), Italy (40), the UK, and Germany (34), while higher rates were found in New Zealand (35), Canada (48), and the USA (54, 55). Because rapid changes in a country’s health care system, poor distribution of staff and funds, and difficulties in psychiatric training are supposed to enhance burnout (56), the low rates found in this study could be explained by the relatively low occurrence of these factors in the Swiss medical system over the last decade. Another explanation could be the high quality of life that Switzerland is known for (57), given that lifestyle aspects can be protective against burnout (56). This study also confirms that greater age (56, 58) is associated with lower burnout risk. While attendant psychotherapy for patients as well as intense cooperation could be protective factors against burnout, it has to be taken into account that the latter can also be experienced as a stressful event.

As in other studies, we found strong correlations between high job satisfaction and low burnout (23, 46). This emphasizes the importance of focusing on both indicators while aiming at higher sustainability in health care systems. Thus, enhancing job satisfaction may assist in strengthening psychiatrists who score high on various dimensions of burnout (59). Similarly, a reduction of burnout risk may prevent unsatisfied psychiatric staff.

Compared to other highly developed countries, in Switzerland, the three explored indicators of sustainability scored high, suggesting a long lasting, persistently functioning outpatient mental health care system. To maintain and foster cooperation, regional networks should be implemented by providing leadership, time for cooperation, reimbursement for meetings, and integration of interdisciplinary cooperation practices in the curricula of medical students and residents in psychiatry (29, 41). Integrated care models that promote community and trans-sectoral health care should be embedded in national health care systems (60). Job satisfaction and burnout among psychiatrists should be a central focus while developing health care concepts that also consider practice setting, attendant psychotherapy for patients, networking, quantity, and duration of cooperation and, in particular, strengthening the quality of cooperation.

### Limitations

Results of this study may be biased because only German-speaking psychiatrists could be included in the study. These results need to be confirmed by additional investigations in other parts of the country. The response rate of 23.7% might have arisen because neither follow-up with non-respondents nor reimbursement for study participation was possible. This major limitation narrows the informative value of the present data because we lack information about the group of non-respondents. Because our survey does not explain variables that could not have been measured, additional research including additional predictive variables is recommended. Despite these limitations, our study has important implications for health care policy makers, health care educators, and future psychiatrists and can help to foster sustainability in outpatient mental health care.

## Conflict of Interest Statement

The research presented was conducted in the absence of any commercial or financial relationships that could be construed as potential conflicts of interest.
